# Vitamin E TPGS-Poloxamer Nanoparticles Entrapping a Novel PI3Kα Inhibitor Potentiate Its Activity against Breast Cancer Cell Lines

**DOI:** 10.3390/pharmaceutics14091977

**Published:** 2022-09-19

**Authors:** Suhair Sunoqrot, Sundos Aliyeh, Samah Abusulieh, Dima Sabbah

**Affiliations:** Department of Pharmacy, Faculty of Pharmacy, Al-Zaytoonah University of Jordan, Amman 11733, Jordan

**Keywords:** breast cancer, nanomedicine, PI3Kα, poloxamers, polymeric nanoparticles, TPGS

## Abstract

N-(2-fluorphenyl)-6-chloro-4-hydroxy-2-quinolone-3-carboxamide (R19) is a newly synthesized phosphatidylinositol 3-kinase alpha (PI3Kα) inhibitor with promising activity against cancer cells. The purpose of this study was to develop a polymeric nanoparticle (NP) formulation for R19 to address its poor aqueous solubility and to facilitate its future administration in preclinical and clinical settings. NPs were prepared by nanoprecipitation using two polymers: D-α-tocopheryl polyethylene glycol 1000 succinate (vitamin E TPGS) and the poloxamer Pluronic P123 in different ratios. Physicochemical characterization of the NPs revealed them to be around 100 nm in size with high monodispersity, a spherical morphology, and an almost neutral surface charge. The NPs achieved ~60% drug loading efficiency and sustained release of R19 for up to 96 h, with excellent colloidal stability in serum-containing cell culture media. NPs containing TPGS enhanced R19’s potency against MCF-7 and MDA-MB-231 breast cancer cells in vitro, with half-maximal inhibitory concentrations (IC_50_) ranging between 1.8 and 4.3 µM compared to free R19, which had an IC_50_ of 14.7–17.0 µM. The NPs also demonstrated low cytotoxicity against human dermal fibroblasts and more significant induction of apoptosis compared to the free drug, which was correlated with their cellular uptake efficiency. Our findings present a biocompatible NP formulation for the delivery of a cancer-targeted PI3Kα inhibitor, R19, which can further enhance its potency for the treatment of breast cancer and potentially other cancer types.

## 1. Introduction

Despite advances in early detection and medical treatments, cancer remains one of the leading causes of death globally [1]. Cancer treatment regimens typically include surgery, radiotherapy, and chemotherapy [2]. Chemotherapy involves the use of cytotoxic agents, which are accompanied by immediate and chronic side effects that can significantly impact cancer patients’ quality of life. The side effects are mainly attributed to the systemic distribution of the cytotoxic agent and a lack of selectivity in killing normal and cancer cells alike [3]. For this reason, significant efforts have been dedicated to the discovery of targeted therapies that can reduce the systemic side effects of chemotherapy.

Protein kinases are important enzymes that modify other proteins by phosphorylation, which consequently regulates the function of these proteins and their downstream signaling pathways. Several kinases have been found to be dysregulated in various cancers, which has inspired the development of kinase inhibitors as cancer-targeted therapies [4]. The phosphatidylinositol-3-kinases (PI3Ks) are a family of lipid kinases that are important for the regulation of cell metabolism, proliferation, migration, and survival [5]. PI3Ks are part of a signaling pathway that includes Akt and the mammalian target of rapamycin (mTOR). The PI3K/Akt/mTOR signaling pathway regulates the growth of both normal and cancer cells and has been shown to be hyperactivated in cancer cells [6,7,8]. Three classes of PI3Ks are known; class I, II, and III. Class I can be divided into Class I_A_ and Class I_B_. Class I_A_ is the class of concern since it is directly linked to cancer progression. Class I_A_ PI3K consists of α, β, and δ isoforms, which are encoded by the genes PIK3CA, PIK3CB, and PIK3CD, respectively [5,9]. Several cancers have demonstrated abnormal PI3Kα/Akt/mTOR signaling pathway activation, in which PIK3CA gene expression has been implicated [9].

**Figure 1:**
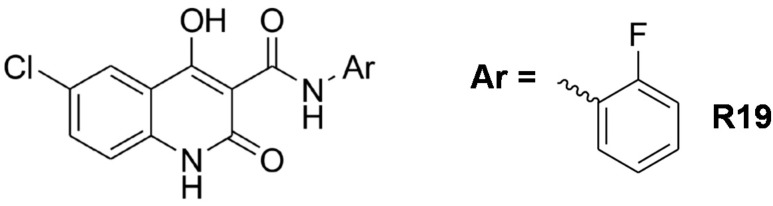
Chemical structure of the N-phenyl-6-chloro-4-hydroxy-2-quinolone-3-carboxamide series and R19 [9].

N-phenyl-6-chloro-4-hydroxy-2-quinolone-3-carboxamides (Figure 1) are a group of newly developed anticancer agents with inhibitory activity against PI3Kα. A series of different analogues were recently synthesized to achieve the maximum inhibition of cancer cells with the lowest possible toxicity on normal cells. N-(2-fluorphenyl)-6-chloro-4-hydroxy-2-quinolone-3-carboxamide (R19) was found to be an effective inhibitory agent against human colon carcinoma (HCT-116) and human epithelial colorectal adenocarcinoma (Caco-2) cell lines, with half-maximal inhibitory concentrations (IC_50_) of 5.3 and 17.0 µM, respectively. Moreover, R19 was tested for its activity on the PI3K/Akt/mTOR signaling pathway, where gene expression analysis revealed a significant reduction in the expression of PI3K and Akt genes and a significant increase in the pro-apoptotic gene Bcl-2-associated death promoter (BAD) [9]. Despite its promising anticancer potency, R19 and its newly developed analogs suffer from poor aqueous solubility, which will be a limiting factor in the clinical translation of this class of compounds.

Nanotechnology has revolutionized the way that we treat diseases, especially cancer. Nanosized drug carriers or nanoparticles (NPs) can improve the solubility, permeability, absorption, delivery, and targeting of drug molecules to the site of action [10,11,12]. Polymeric NPs are a particularly attractive delivery approach due to their highly tunable properties, which can be leveraged to achieve better solubility, bioavailability, controlled release, and targeting of drug candidates by passive and active targeting mechanisms [13,14,15,16].

Poloxamers are amphiphilic triblock copolymers composed of poly(ethylene oxide) (PEO) and poly(propylene oxide) (PPO) (PEO-PPO-PEO) [17,18]. They are biocompatible polymers that are used in various applications, especially as nanocarriers for hydrophobic drugs [19]. Poloxamers have also been combined with other polymers to improve drug loading, impart NP stability, and enhance drug solubility [18]. On the other hand, D-α-tocopheryl polyethylene glycol 1000 succinate (vitamin E TPGS or TPGS) is a water-soluble form of vitamin E that is used in drug delivery to prolong the circulation half-life, enhance the bioavailability, improve the cytotoxicity, and synergize with other anticancer drugs [20,21,22,23]. TPGS is used as a nanocarrier for hydrophobic and poorly water-soluble drugs due to its amphiphilic nature [24,25].

In this study, we hypothesized that two biocompatible polymers, TPGS and a poloxamer (Pluronic P123), can be used as nanocarriers for R19 encapsulation and delivery, combining the benefits of both TPGS and poloxamers. To verify our hypothesis, R19 NPs at different ratios of TPGS and Pluronic P123 were prepared and characterized for their physicochemical properties. The NPs were also tested on breast cancer cell lines to evaluate their bioactivity and biocompatibility.

## 2. Materials and Methods

### 2.1. Materials

N-(2-fluorphenyl)-6-chloro-4-hydroxy-2-quinolone-3-carboxamide (R19) was synthesized and characterized as previously described [9]. Pluronic P123 (P123), TPGS, and Nile red (NR) were purchased from Sigma-Aldrich (St. Louis, MO, USA). Phosphate buffered saline solution (PBS, 10×, pH 7.4), potassium bromide (KBr), and dimethyl sulfoxide (DMSO) were procured from Fisher Chemicals (Pittsburgh, PA, USA). Spectra/Por dialysis membranes with 3500 and 12,000–14,000 Da molecular weight cut-offs (MWCO) were obtained from Repligen (Waltham, MA, USA). Ultrapure water was prepared using a Millipore Direct-Q 5UV system (EMD Millipore, Burlington, MA, USA).

### 2.2. Preparation of R19-Loaded NPs

**Figure 2:**
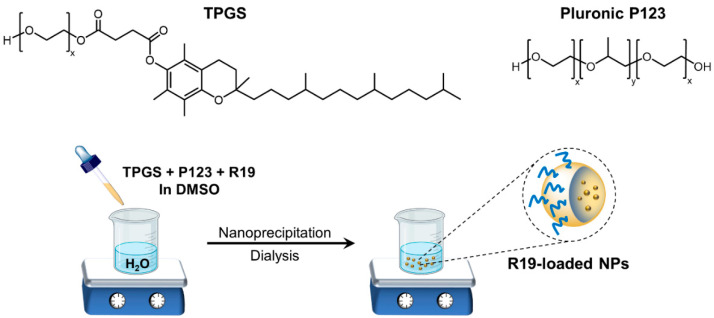
Structures of TPGS and P123 and a schematic of R19 NP preparation by nanoprecipitation.

R19-loaded NPs were produced via the nanoprecipitation technique, as previously described [26]. Different NP formulations were prepared by varying the P123: TPGS ratio (Table 1), and each batch was prepared in triplicate. Stock solutions of each polymer (P123 and TPGS) were first prepared at 20 mg/mL in DMSO. Each batch consisted of a 20 mg polymer or polymer mixture, and the amount of drug was fixed at 2 mg. The organic phase was prepared by mixing the appropriate volumes of polymer solutions in a microtube (total volume 1 mL), to which 2 mg of R19 powder was added, and the mixture was vortexed until the drug was completely dissolved. The resultant organic phase was then added dropwise to 10 mL of ultrapure water under continuous stirring. After 30 min of stirring, the NP dispersion was transferred to a dialysis membrane (3500 MWCO) and was dialyzed against 2 L of deionized water under gentle stirring for 24 h. After 24 h, the dialysis bag was emptied into a vial, and the samples were either used directly or were stored at 4 °C for further characterization. Blank NPs were prepared as described above but without the addition of R19. NR-labeled NPs were prepared by replacing R19 with NR (0.2 mg per NP formulation), and the NPs were purified by dialysis, as described above. A schematic of the NP preparation process is presented in Figure 2.

### 2.3. Particle Size and Zeta Potential Measurements

Dynamic light scattering (DLS) was used for the measurement of the particle size and polydispersity index (PDI) of the NPs. Electrophoretic light scattering was used for zeta potential measurements. Samples were analyzed using a Nicomp^®^ Nano Z3000 instrument (Entegris, Billerica, MA, USA). For the analysis, fresh NPs (200 µL) were diluted at a ratio of 1:1 with ultrapure water to obtain a scattering intensity between 200 and 300 kHz. Results were expressed as the mean ± standard deviation (SD), and each formulation was prepared in triplicate.

### 2.4. Drug Loading Efficiency Determination

The amount of R19 loaded in the NPs and the drug loading efficiency were determined by UV-Vis (UV-1800 spectrophotometer, Shimadzu, Kyoto, Japan). Fresh NPs (100 µL) were diluted 10× in DMSO, and their UV absorbance was measured at 309 nm (λ_max_ for R19). The concentration of loaded R19 was then calculated based on a calibration curve of drug absorbance at 309 nm versus concentration in DMSO. The concentration was multiplied by the total volume of each formulation to obtain the total amount of loaded drug; then, the drug loading efficiency (DL%) was calculated according to Equation (1):

Drug loading efficiency (DL%) = (Actual amount of drug loaded)/(Initial amount of drug added) × 100%(1)

NR-labeled NPs were also diluted 10× in DMSO, and the absorbance was measured at 552 nm. A calibration curve of NR in DMSO was prepared at 552 nm and was used to determine the NR concentration in the labeled NPs.

### 2.5. Transmission Electron Microscopy (TEM) Imaging

R19-loaded NPs were visualized by transmission electron microscopy (TEM). Samples were prepared and were imaged as previously described [27].

### 2.6. Characterization by Fourier Transform-Infrared (FTIR) Spectroscopy

For analysis by FTIR, fresh R19-loaded NPs were lyophilized (FreeZone 4.5 L, Labconco, Kansas City, MO, USA) and transformed into KBr pellets. The FTIR spectra of R19, Pluronic P123, TPGS, and the R19-loaded NPs were acquired using an IR Affinity-1 spectrometer (Shimadzu, Kyoto, Japan) within a wavenumber range of 3600–500 cm^−1^.

### 2.7. Differential Scanning Calorimetry (DSC)

The thermal transitions of the R19-loaded NPs were examined using a Netzsch DSC 204 F1 instrument (Selb, Germany). Approximately 10 mg of lyophilized NPs, individual polymers, and R19 were used for the analysis. The samples’ thermal behavior was investigated by heating from ambient temperature to 250 °C at a rate of 10 °C/min under a nitrogen atmosphere.

### 2.8. Colloidal Stability of R19-Loaded NPs

The stability of the prepared NPs was evaluated upon incubation in cell culture media for 24 h at 37 °C by monitoring the changes in particle size and in the PDI. In total, 200 µL of each sample were diluted with 200 µL of cell culture medium supplemented with 10% fetal bovine serum (FBS), and the samples were incubated for 24 h with shaking at 100 rpm and 37 °C (Biosan ES-20 Orbital Shaker-Incubator, Riga, Latvia). Particle size and PDI of the NPs were measured before and after 24 h incubation by DLS. The experiment was performed in triplicate, and the results were reported as the mean ± SD.

### 2.9. In Vitro Release of R19 from R19-Loaded NPs

The in vitro release of R19 was evaluated using the dialysis method. Briefly, 1-mL aliquots of fresh NPs were transferred to a dialysis bag (12,000–14,000 MWCO) in triplicate and were inserted into vials containing 30 mL of phosphate-buffered saline (PBS, pH 7.4) supplemented with 0.5% *w*/*v* Tween 80 to maintain sink conditions [25]. The samples were incubated under shaking at 100 rpm and 37 °C (Biosan ES-20 Orbital Shaker-Incubator). At the designated sampling time points, 10 mL of each release medium was withdrawn and replaced with 10 mL of fresh buffer. At the 24, 48, 72, and 96 h time points, 10 mL samples were withdrawn, and the remaining release medium was replaced with 30 mL of fresh buffer. The collected samples were lyophilized, redissolved in 1 mL of DMSO, and centrifuged for 5 min at 4000 rpm to precipitate the undissolved buffer salts. The amount of R19 released was then measured by analyzing the UV absorbance of the supernatant based on the calibration curve of R19 in DMSO. Results were expressed as the average cumulative release (%) versus time (h). The release profiles were compared by calculating the similarity factor (*f*_2_) between each of the two formulations according to Equation (2) [28]:

*f*_2_ = 50 × log[(1 + 1/*n* ∑(*R_t_* – *T_t_*)^2^)^−0.5^ × 100] (2)

where *n* is the number of sampling time points, and *R_t_* and *T_t_* are the cumulative amounts released from every two formulations at each time point *t*.

### 2.10. Cell Viability Assays

Cell viability assays were conducted on the MCF-7 and MDA-MB-231 breast cancer cells as well as on human dermal fibroblasts (HDFs) as normal cells. Cell lines were obtained from the American Type Culture Collection (ATCC, Manassas, VA, USA). MCF-7 cells were grown in Minimum Essential Medium (MEM; Gibco, Thermo Fisher Scientific, Waltham, MA, USA). MDA-MB-231 cells were grown in Roswell Park Memorial Institute (RPMI) 1640 medium (Euroclone SpA, Milan, Italy). HDFs were grown in Dulbecco’s Modified Eagle’s Medium (DMEM; Euroclone SpA). All media were supplemented with 10% heat-inactivated fetal bovine serum (FBS; Capricorn Scientific, Ebsdorfergrund, Germany) and penicillin–streptomycin (100 U/mL–100 µg/mL; Euroclone SpA). Cells were maintained at 37 °C in a humidified 5% CO_2_ incubator. For the viability assays, cells were seeded in 96-well plates at a density of 10,000 cells/well (MCF-7 and MDA-MB-231) or 7000 cells/well (HDFs) and were incubated overnight. The next day, cells (*n* = 5 per treatment group) were treated with R19 (from a 20 mg/mL stock solution in DMSO) and R19-loaded NPs (freshly prepared and dispersed in water) at concentrations between 0 and 300 µM of R19 diluted in complete culture medium for 48 h. Another group of cells was treated with blank NP formulations at concentrations equivalent to the concentrations used for R19-loaded NPs. At the end of the incubation period, the medium was removed, and 100 µL of fresh medium containing 0.5 mg/mL of 3-(4,5-dimethylthiazol-2-yl)-2,5-diphenyltetrazolium bromide (MTT, Wheeling, IL, USA) was added to each well. Following incubation for 2 h, the medium was removed, and 100 µL of DMSO was added to each well to dissolve the formazan crystals. The optical density of each well was measured at 540 nm using a BioTek Synergy HTX Multi-Mode Reader. Cell viability was expressed as the % relative to the untreated cells. IC_50_ values were determined by a non-linear regression analysis of cell viability versus concentration curves using GraphPad Prism software version 7.00 (San Diego, CA, USA).

### 2.11. Mitochondrial Membrane Potential Assay

The mitochondrial membrane potential of MCF-7 and MDA-MB-231 cells treated with R19 and the R19-loaded NPs was measured using the JC-1 probe (Abcam, Cambridge, UK) [29]. Briefly, cells were seeded in 96-well plates at a density of 10,000 cells/well overnight. The next day, cells were treated with 20 µM of free R19 or an equivalent concentration of R19-loaded NPs for 24 h in triplicate (100 µL per well). After 24 h, JC-1 was diluted in PBS to a concentration of 20 µM, and 100 µL of the dye solution was added to each well. The cells were incubated with JC-1 for 10 min at 37 °C, and the fluorescence intensity of the wells was then measured using a BioTek Synergy HTX Multi-Mode Reader at 485 nm/528 nm excitation/emission wavelengths for the detection of the green JC-1 monomers and at 540 nm/620 nm excitation/emission wavelengths for the detection of the red JC-1 aggregates. The results were expressed as the ratio of green/red fluorescence after subtracting the background fluorescence of the unstained cells.

### 2.12. Cellular Uptake of NR-Labeled NPs

Cellular uptake assays were conducted in MCF-7 and MDA-MB-231 cells using NR-labeled NPs. Briefly, cells were seeded in 24-well plates at a density of 50,000 cells/well. The next day, cells were treated with NR dissolved in complete culture medium (MEM for MCF-7 cells or RPMI 1640 for MDA-MB-231 cells) at a concentration of 1 µg/mL or at an equivalent concentration of NR NPs for 1 h (*n* = 3). Then, the medium was removed, the cells were washed with PBS twice, and 500 µL of PBS was added to each well. NR fluorescence was quantified using a BioTek Synergy HTX Multi-Mode Reader at 540 nm/620 nm excitation/emission wavelengths. Untreated cells were used as background controls, and cell-associated fluorescence was normalized relative to the untreated cells.

### 2.13. Statistical Analysis

All data are presented as mean ± SD of at least three different trials. Differences between sample means were compared using one- or two-way analysis of variance (ANOVA) followed by Tukey’s or Sidak’s multiple comparison test, respectively, where a *p*-value of 0.05 was considered statistically significant. All analyses were performed in GraphPad Prism version 7.00.

## 3. Results and Discussion

### 3.1. Preparation and Characterization of R19-Loaded NPs

Here, we investigated the feasibility of incorporating R19 in a polymeric NP formulation to ensure its solubility, stability, and potential targeting ability when applied in vivo due to the enhanced permeability and retention (EPR) effect [15,30]. NPs were prepared by nanoprecipitation, as it is a simple and convenient method for entrapping hydrophobic drugs in polymeric NPs (Figure 2) [31]. R19 was formulated using two types of biocompatible polymers to evaluate the loading capacity of the NPs for the drug, the size of the NPs produced, and the effectiveness and safety toward cancer and normal cells.

**Figure 3:**
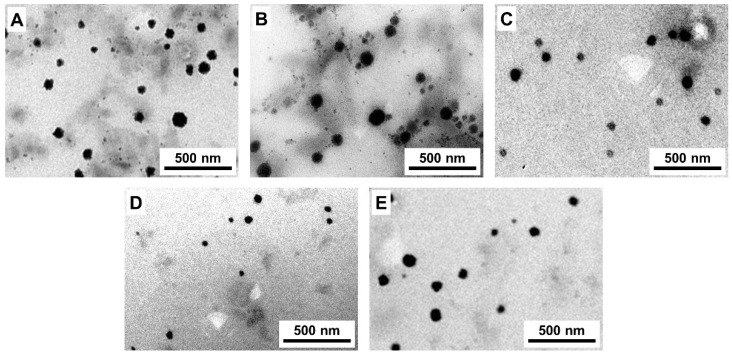
TEM images of R19-loaded NPs prepared using (**A**) 100% TPGS, (**B**) 25% P123/75% TPGS, (**C**) 50% P123/50% TPGS, (**D**) 75% P123/25% TPGS, and (**E**) 100% P123.

Several formulations were prepared using different ratios of P123: TPGS (Table 1), and the characteristics of the NPs in terms of particle size, PDI, and DL% are presented in Table 2. The R19-loaded NPs were initially prepared using 100% P123. The average DL% was 59%, and the average particle size of the NPs was 100 nm, with a PDI of 0.17. Then, TPGS was used in combination with P123 to evaluate its effect on the DL% and particle size. Different ratios of both polymers were prepared: 100% TPGS (0% P123), 25% P123/75% TPGS, 50% P123/50% TPGS, and 75% P123/25% TPGS. Interestingly, as shown in Table 2, the different NP formulations showed similar average particle sizes ranging between 92 and 112 nm. With respect to the DL%, it ranged between 57 and 61%. Overall, the different ratios of P123 with TPGS did not reveal a significant difference with regard to the DL% nor the particle size of the NPs obtained. Likewise, the PDI of the formulations was relatively small and ranged between 0.14 and 0.20. PDI is a measure of the uniformity of the particle size distribution [32]. The PDI results revealed a narrow particle size distribution in all of the NP formulations as the values were close to 0, reflecting the excellent monodispersity of the NPs. Furthermore, the NPs were characterized by a spherical morphology when observed under TEM (Figure 3).

Zeta potential is used as an indicator of a colloidal dispersion’s stability that is based on the surface charge of the particles in contact with water [33]. The zeta potential values for all of the NP formulations ranged between −30 mV and +30 mV, indicating that the NPs carried an almost neutral surface charge. Since this may affect their aggregation tendency, particularly when in contact with biological fluids, this led us to investigate the stability of the R19-loaded NPs in cell culture media, as described later.

**Figure 4:**
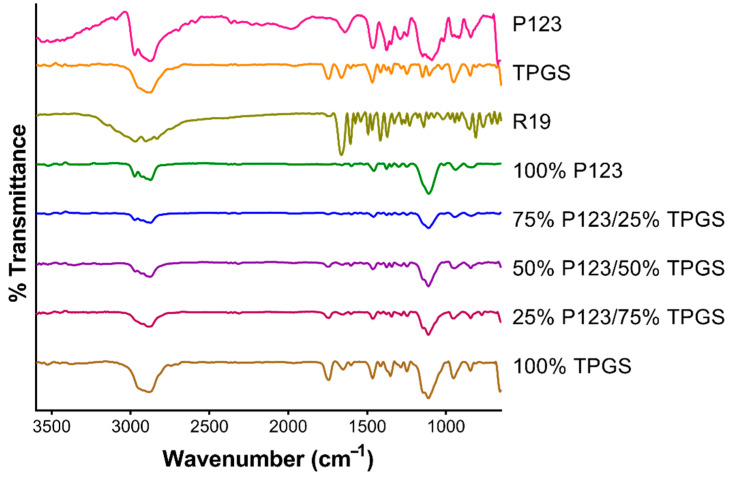
FTIR spectra of R19, P123, TPGS, and R19-loaded NPs.

The five NP formulations were characterized by FTIR spectroscopy and were compared to free R19 and the individual polymers P123 and TPGS (Figure 4). TPGS displayed characteristic bands at 1750 cm^−1^ and at 2900–3000 cm^−1^, corresponding to the O−C=O stretching of its ester groups and C−H stretching, respectively [34]. The O−C=O stretching band appeared in the R19 NP formulation prepared with 100% TPGS and in the other formulations containing TPGS but with lower intensities due to the lower concentration. Moreover, this band disappeared in the formulations containing 100% P123. Pluronic P123 is a polyether characterized by a broad C−O−C stretching band between 1000 and 1100 cm^−1^ [35]. P123 also showed a C−H stretching band at 2900–3000 cm^−1^, which is related to asymmetric C−H stretching in the methyl groups in PPO in addition to the band at 1300–1400 cm^−1^ [36]. R19 NPs exhibited the same bands at 1300–1400 cm^−1^. R19 and TPGS showed similar bands that overlapped with P123. In addition to a broad –OH stretching band between 2600 and 3200 cm^−1^, R19 displayed a sharp band at 1660 cm^−1^ corresponding to the amide C=O stretching. This band was present in the NP formulations but had a weaker intensity, most likely due to the low % of R19 in the formulations (around 6% *w*/*w*). Overall, no new peaks were formed, which indicated no drug–excipient interaction.

**Figure 5:**
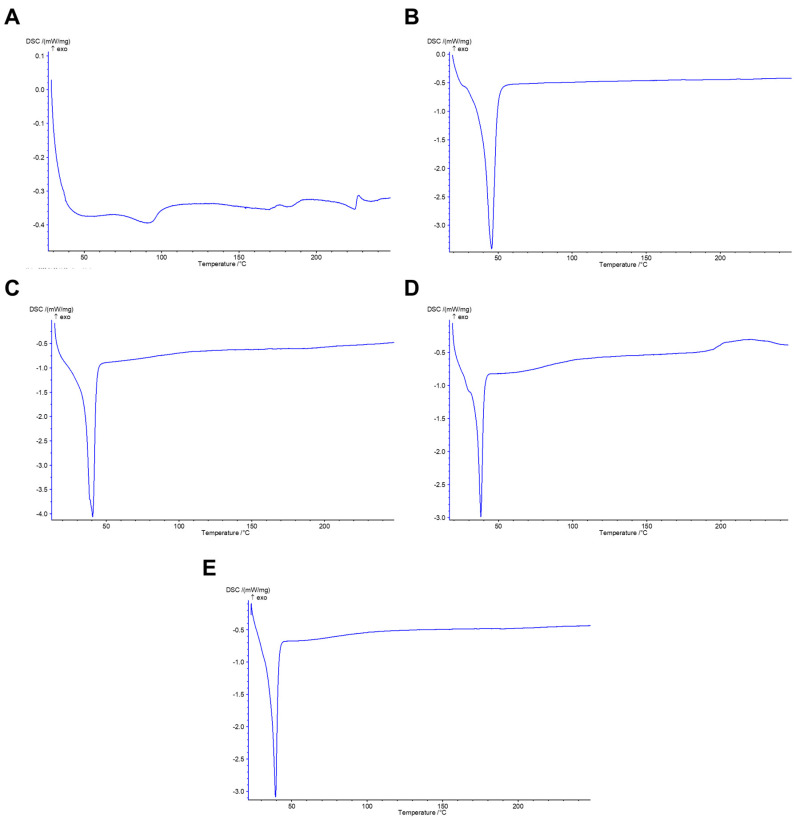
DSC thermograms of (**A**) R19, (**B**) neat TPGS, and R19-loaded NPs composed of (**C**) 100% TPGS, (**D**) 25% P123/75% TPGS, and (**E**) 50% P123/50% TPGS.

The thermal transitions of the NP formulations were examined by DSC. As shown in Figure 5A, R19 displayed broad endothermic melting peaks between 80 and 100 °C and between 150 and 270 °C, indicating the nearly amorphous nature of the compound. Neat TPGS (Figure 5B) exhibited a sharp endothermic melting peak at around 45.9 °C, which is close to the literature value [37]. The R19-loaded NPs prepared using 100% TPGS (Figure 5C) showed a reduction in the TPGS melting peak to 40.9 °C, suggesting an interaction between the drug and the polymer, most likely representing van der Waals and potentially H-bonding interactions. As P123 was added to the formulation, the melting peak of TPGS was slightly lowered to 38.2–39.5 °C in the R19 NPs composed of 25% P123/75% TPGS (Figure 5D) and 50% P123/50% TPGS (Figure 5E), reflecting additional polymer–polymer and polymer–drug interactions. Notably, the peaks that were originally present in R19 disappeared in all of the NP formulations, suggesting that it is contained in the NPs in an amorphous state. The neat P123 and NPs with 75% and 100% P123 could not be analyzed due to the liquification of the polymer upon heating and the technical limitations of the DSC instrument. However, according to the literature, P123 is known for its thermal stability, with a weak endothermic peak appearing at 75 °C corresponding to the melting of PEO chains and a strong endothermic peak at around 400 °C due to polymer decomposition [38].

### 3.2. Stability of R19-Loaded NPs

**Figure 6:**
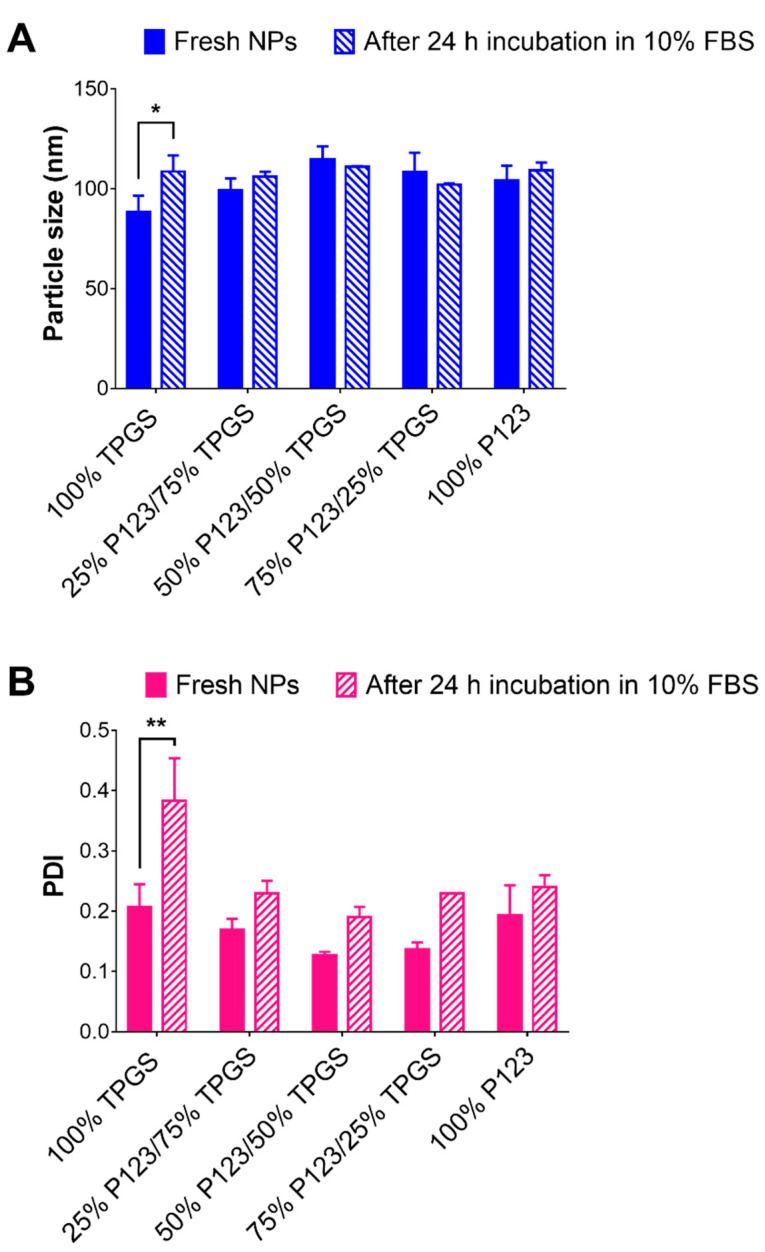
Change in (**A**) particle size and (**B**) PDI of R19-loaded NPs after 24 h incubation in cell culture medium supplemented with 10% FBS at 37 °C. Results are presented as the mean ± SD (*n* = 3). * *p* < 0.05 and ** *p* < 0.01 based on two-way ANOVA followed by Sidak’s multiple comparison test.

As mentioned earlier, the R19-loaded NPs may have a tendency toward aggregation due to their low surface charge. It is challenging to maintain a stabilized particle size distribution of the NPs in a colloidal dispersion, particularly in biological fluids. Since the NPs were going to be evaluated in in vitro cell culture, we tested the colloidal stability of the NPs in a serum-supplemented cell culture medium for 24 h. Figure 6 represents the particle sizes of the samples before and after 24 h of incubation at 37 °C in the cell culture medium. Interestingly, all of the NPs, except for those prepared using 100% TPGS, showed no significant changes in the particle size nor in the PDI upon incubation. On the other hand, the NPs composed of 100% TPGS underwent a significant size increase (*p* < 0.05) from 88 nm to 108 nm that was accompanied by a marked increase in the PDI from 0.21 to 0.38 (*p* < 0.01). These results strongly indicate that the presence of P123, even at small percentages, can make the NPs more stable against aggregation when applied in biological systems.

### 3.3. In Vitro Release of R19 from R19-Loaded NPs

**Figure 7:**
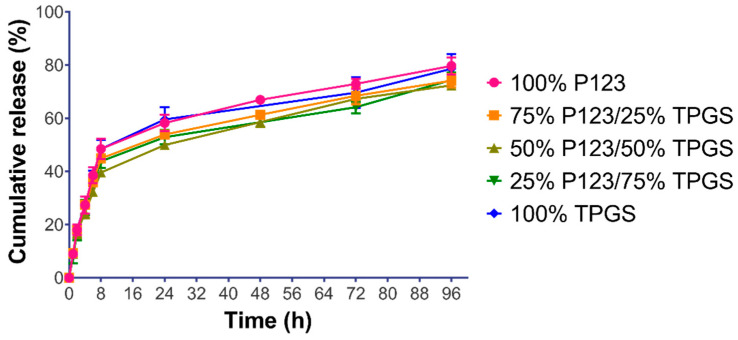
In vitro release of R19 from R19-loaded NPs in PBS pH 7.4 at 37 °C, demonstrating sustained drug release up to 96 h. Results are expressed as the mean cumulative release % ± SD (*n* = 3) plotted against time (h).

The different formulations of the R19-loaded NPs were subjected to release testing in PBS buffer with a pH of 7.4 at 37 °C to mimic physiological conditions. As shown in Figure 7, the cumulative drug release from all of the formulations was almost superimposable. All of the profiles were characterized by a biphasic release pattern, with a relatively fast release phase within the first 8 h followed by a more sustained release phase up to 96 h. The first phase may be related to the drug release from the surface of the NPs, whereas the second phase may be attributed to the slow diffusion of the drug from inside the NPs. Within the first 8 h of incubation, the cumulative drug release across all of the formulations was between 40 and 49%. After 24 h, the cumulative drug release increased more gradually to 50–58%. The same trend was observed at 48 and 72 h. After 96 h, the cumulative drug release reached 72−79% among the various formulations. Our results are in line with previous work on similar polymeric systems. For example, TPGS-formulated liposomes or NPs have been studied to determine in vitro release and showed a relatively slow release and a cumulative release of 82% after 48 h [39,40]. As for P123-based nanoformulations, the release of different drugs was affected by the percentage of P123 and ranged between 50 and 80% [41]. On the other hand, formulations with P123/TPGS mixtures showed a slow in vitro release that ranged between 40 and 50%, which was believed to be caused by the micelles formed from P123/TPGS and the preference of the hydrophobic drug to remain in the hydrophobic core of the micelles due to its low solubility in the release medium [42]. The similarity factor (*f*_2_) was calculated between the various NP formulations to examine the differences between the release profiles. As shown in Table 3, *f*_2_ was greater than 50 in all of the formulations, confirming their similar release behavior [28].

### 3.4. Cell Viability Assays

**Figure 8:**
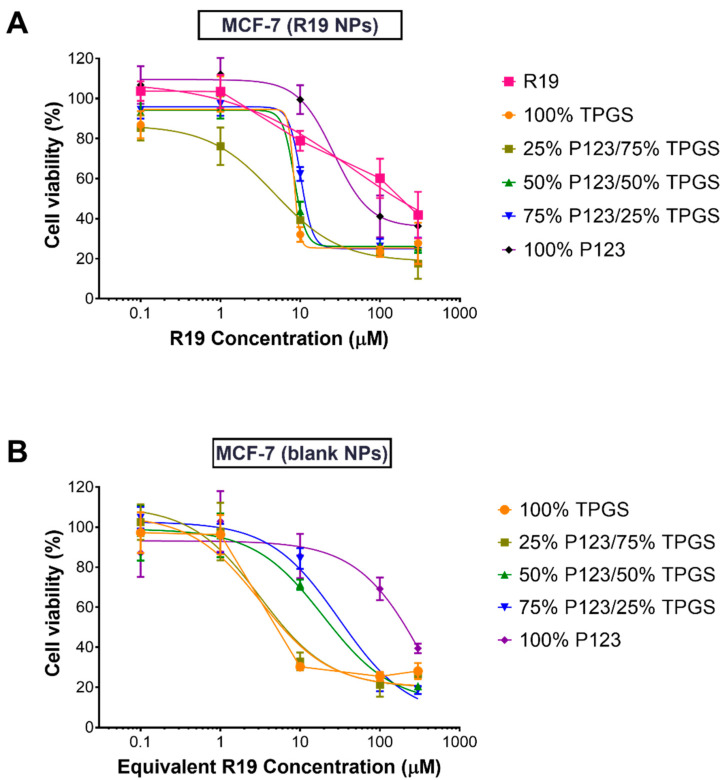
Percentage viability of MCF-7 cells treated with (**A**) R19 and R19-loaded NPs and (**B**) blank NPs for 48 h (*n* = 5).

**Figure 9:**
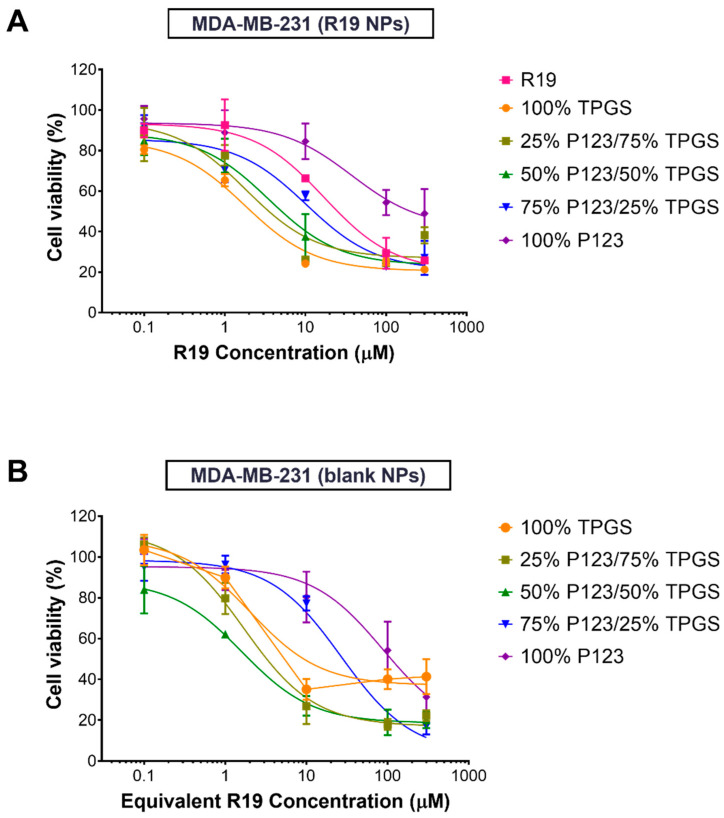
Percentage viability of MDA-MB-231 cells treated with (**A**) R19 and R19-loaded NPs and (**B**) blank NPs for 48 h (*n* = 5).

The anticancer activity of R19 and R19-loaded NPs was evaluated against two breast cancer cell lines: MCF-7 and MDA-MB-231, because breast cancer is the most common malignancy affecting women and accounts for the highest percentage of cancer-related deaths [43]. Cells were treated with increasing concentrations of R19 and the NPs for 48 h, followed by the MTT assay. As shown in Figure 8A and Figure 9A, all of the treatments caused a dose-dependent inhibition of cell growth, but with varying degrees. The dose–response curves were fitted by non-linear regression analysis to obtain the IC_50_ values, which correspond to the treatments’ potency. As shown in Table 4, R19 displayed similar potency against both cell lines, with IC_50_ values of 14.7 and 17.0 µM in the MCF-7 and MDA-MB-231 cells, respectively. These values were similar to the R19 potency in colorectal cancer cells [9]. Since the PI3K/Akt/mTOR pathway is dysregulated in these cancer cell lines, it was expected that R19 would show similar inhibitory activity. The potent anticancer effect of R19 in breast cancer cell lines strongly support its application as a potential targeted therapy in hormone receptor-positive and triple-negative breast cancer.

As for the R19 NP formulations, lower IC_50_ values were obtained compared to the free drug in both cell lines, with the exception of 100% P123 NPs. The highest potency was observed for the R19 NPs prepared using 100% TPGS and 25% P123/75% TPGS in both the MCF-7 and MDA-MB-231 cells, with almost a three-fold reduction in the IC_50_ values in MCF-7 cells and a two- to nine-fold reduction in MDA-MB-231 cells. Generally, the IC_50_ value increased as the % of TPGS decreased, and the NPs with 100% P123 displayed the highest IC_50_ values and the poorest fit to the dose–response curve (*R*^2^ < 0.9). The enhancement of anticancer potency in the NP formulations can likely be attributed to the polymeric carriers, particularly TPGS. TPGS has been reported to inhibit cancer cell proliferation through cell cycle arrest and the induction of apoptosis [44,45]. In addition, TPGS has been shown to overcome cancer drug resistance by downregulating the expression of P-glycoprotein efflux pumps [46,47]. These findings were confirmed by testing equivalent concentrations of the NPs without the drug under the same conditions. As shown in Table 4, blank NPs containing more than 50% TPGS were almost equally potent as the drug-loaded formulations. However, as the % of P123 increased, a reduction in potency was observed, similar to the drug-loaded NPs. These results provide a promising approach for the application of TPGS-containing R19 NPs, not only in breast cancer but also in multidrug-resistant cancer types.

**Figure 10:**
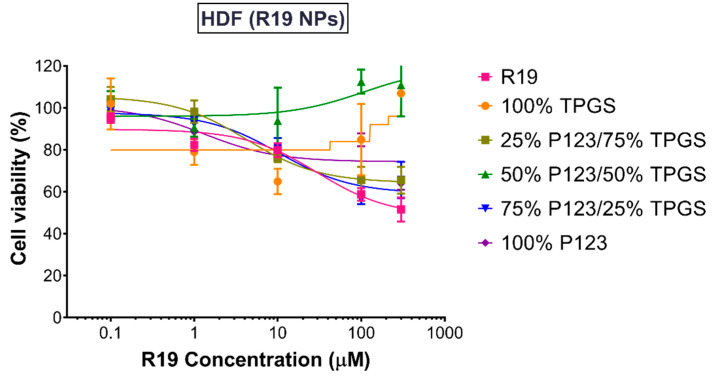
Percentage viability of HDF treated with R19 and R19-loaded NPs for 48 h (*n* = 5).

Having shown excellent activity against cancer cells, equivalent concentrations of R19-loaded NPs were also tested in HDF to determine their selectivity and biocompatibility. As depicted in Figure 10, the NPs exhibited more than 60% cell viability at the highest concentration tested (300 µM), whereas the free drug caused an almost 50% reduction in cell viability at the same concentration. Moreover, the NPs prepared using 100% TPGS and 50% P123/50% TPGS maintained a cell viability of ~100% at the highest concentration, indicating their superior biocompatibility. As all of the groups resulted in more than 50% cell viability, the IC_50_ values could not be accurately determined. Altogether, the results clearly demonstrate the selectivity of R19 in killing cancer cells, which was enhanced upon incorporation into the NPs. Moreover, the low IC_50_ values obtained for the TPGS-containing NPs in the MDA-MB-231 cells, which represent the more aggressive type of breast cancer, represent a highly promising result for the potential clinical application of the NP formulation as a breast cancer nanomedicine.

### 3.5. Mitochondrial Membrane Potential Assay of R19-Treated Cells

**Figure 11:**
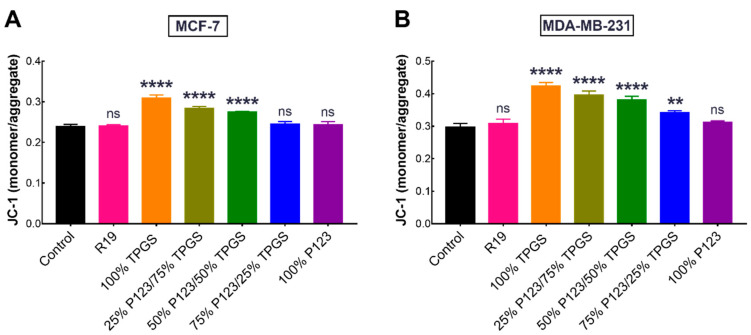
Change in mitochondrial membrane potential of (**A**) MCF-7 and (**B**) MDA-MB-231 cells after 24 h of treatment with 20 µM of R19 and R19-loaded NPs. Results are expressed as the ratio of the green/red fluorescence signals of the JC-1 monomers and aggregates, respectively (mean ± SD; *n* = 3). ** *p* < 0.01, **** *p* < 0.0001, and ns: not significantly different, compared to the control (untreated cells), based on one-way ANOVA followed by Tukey’s multiple comparisons test.

Mitochondrial depolarization is one of the early signs of apoptosis and can be conveniently detected by JC-1. Under normal conditions, JC-1 exists in an aggregated state within the mitochondrial matrix and emits red fluorescence. As the mitochondrial transmembrane potential dissipates in cells undergoing apoptosis, JC-1 is effluxed to the cytoplasm, where it dissociates into monomers that emit green fluorescence. Therefore, the change in the ratio of green/red fluorescence may be used as an indicator of apoptosis induction [48]. As seen in Figure 11A, MCF-7 cells treated with free R19 and R19 NPs composed of 75% P123/25% TPGS and 100% P123 did not show a significant difference in the JC-1 monomer/aggregate ratio compared to the control (untreated cells). Likewise, the mitochondrial membrane potential of MDA-MB-231 cells treated with R19 and R19 NPs composed of 100% P123 was similar to that of the control (Figure 11B). On the other hand, cells treated with the R19 NPs containing TPGS displayed a significantly higher JC-1 monomer/aggregate ratio, signifying that these formulations were able to induce a greater degree of apoptosis compared to the free drug and R19 NPs composed entirely of P123.

### 3.6. Cellular Uptake of NR-Labeled NPs

**Figure 12:**
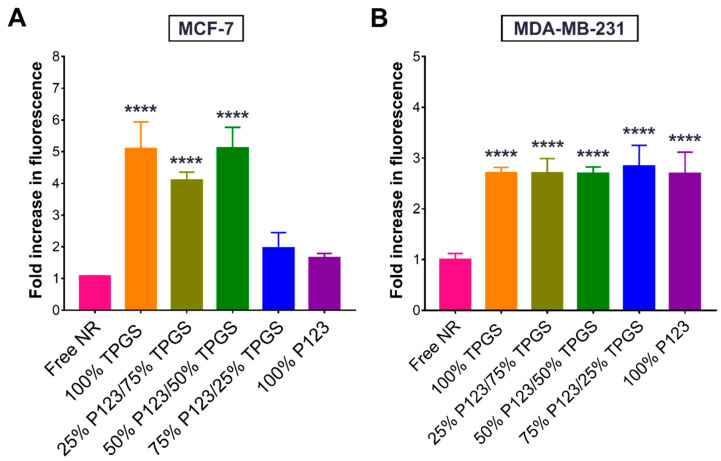
Normalized fluorescence intensities (mean ± SD; *n* = 3) of (**A**) MCF-7 and (**B**) MDA-MB-231 cells treated with free NR or NR-labeled NPs for 1 h. **** *p* < 0.0001 based on one-way ANOVA followed by Tukey’s multiple comparison test.

The observed enhancement in the bioactivity of R19 upon incorporation into the NP formulations may in part be attributed to an enhancement in cellular uptake. To test this hypothesis, R19 was replaced with NR, and the NR-labeled NPs were incubated with the cells. As depicted in Figure 12A, in MCF-7 cells, the NPs composed of high percentages of TPGS (100%, 75%, and 50%) were associated with the highest increase in intracellular fluorescence compared to the free dye, denoting enhanced cellular uptake. As for MDA-MB-231 cells (Figure 12B), all of the NPs exhibited a significant increase in intracellular fluorescence compared to the free dye regardless of their composition. Interestingly, the TPGS-containing NPs achieved a four- to five-fold increase in fluorescence compared to the control in MCF-7 cells, whereas in the MDA-MB-231 cells, the NPs averaged around a 2.7-fold increase in fluorescence. These results are most likely attributed to differences in the membrane permeability between the two cell lines. Nonetheless, the results strongly support the ability of the NP formulations, particularly those containing TPGS, to enhance the cellular uptake of hydrophobic molecules such as R19, consistent with the cell viability and JC-1 assays.

## 4. Conclusions

R19 is a new chemical entity that has been found to be an effective anticancer agent. In this work, R19 was successfully formulated in NPs composed of different combinations of TPGS and Pluronic P123. The NPs had particle sizes of approximately 100 nm and high monodispersity. Additionally, the DL% reached about 60% in the different polymer combinations and all of the NPs sustained the release of the drug up to 96 h. The NPs were highly stable in serum-supplemented cell culture medium, with the exception of the formulations composed of 100% TPGS, which exhibited a tendency for aggregation. Cell viability assays in MCF-7 and MDA-MB-231 breast cancer cell lines revealed an enhanced potency of R19 when it was incorporated into TPGS-containing NPs with reduced cytotoxicity against HDF, most likely by enhancing its cellular uptake and promoting apoptosis. The observed cancer cell selectivity and high biocompatibility of the NP formulations emphasize the positive attributes of the designed polymeric NPs as a promising delivery approach for R19 and its analogs, bringing them one step closer to clinical translation.

## Figures and Tables

**Table 1:** Composition of R19-loaded NPs.

NP	R19 (mg)	Pluronic P123 (mg)	TPGS (mg)
100% TPGS	2	0	20
25% P123/75% TPGS	2	5	15
50% P123/50% TPGS	2	10	10
75% P123/25% TPGS	2	15	5
100% P123	2	20	0

**Table 2:** Characteristics of R19-loaded NPs reported as the mean ± SD of at least three different batches of each NP.

NP	Particle Size * (nm)	PDI	Zeta Potential (mV)	DL%
100% TPGS	108 ± 8	0.20 ± 0.06	−1.7 ± 5.0	60 ± 6
25% P123/75% TPGS	92 ± 7	0.20 ± 0.03	−6.6 ± 19.6	57 ± 7
50% P123/50% TPGS	111 ± 8	0.14 ± 0.01	−5.4 ± 15.3	59 ± 10
75% P123/25% TPGS	112 ± 8	0.15 ± 0.05	16.0 ± 2.9	59 ± 6
100% P123	100 ± 8	0.17 ± 0.02	−10.5 ± 17.1	59 ± 7

* Intensity-weighted.

**Table 3:** Similarity factor (*f*_2_) results for R19-loaded NPs.

Sample 1	Sample 2	*f*_2_
100% P123	75% P123/25% TPGS	71
	50% P123/50% TPGS	60
	25% P123/75% TPGS	66
	100% TPGS	88
75% P123/25% TPGS	50% P123/50% TPGS	75
	25% P123/75% TPGS	84
	100% TPGS	75
50% P123/50% TPGS	25% P123/75% TPGS	78
	100% TPGS	62
25% P123/75% TPGS	100% TPGS	69

**Table 4:** IC_50_ values of R19 and R19-loaded NPs and equivalent concentrations of blank NPs in MCF-7 and MDA-MB-231 cells after 48 h of incubation.

Treatment	IC_50_ (µM; Mean ± SEM *)	
	MCF-7	MDA-MB-231
R19	14.7 ± 5.3	17.0 ± 4.2
100% TPGS	4.3 ± 1.9	1.8 ± 0.4
25% P123/75% TPGS	4.7 ± 1.0	1.8 ± 0.7
50% P123/50% TPGS	4.9 ± 1.1	3.5 ± 1.0
75% P123/25% TPGS	12.3 ± 2.4	10.2 ± 3.2
100% P123	45.4 ± 18.6	37.5 ± 24.3
Blank 100% TPGS	3.2 ± 1.1	2.0 ± 0.8
Blank 25% P123/75% TPGS	3.1 ± 1.0	1.7 ± 0.3
Blank 50% P123/50% TPGS	19.5 ± 4.9	1.6 ± 0.4
Blank 75% P123/25% TPGS	31.0 ± 7.2	26.8 ± 5.8
Blank 100% P123	415.1 ± 448.8	87.1 ± 52.0

* SEM: standard error.

## Data Availability

Not applicable.
